# Functions of HP1 proteins in transcriptional regulation

**DOI:** 10.1186/s13072-022-00453-8

**Published:** 2022-05-07

**Authors:** John M. Schoelz, Nicole C. Riddle

**Affiliations:** Department of Biology, University of Alabama at Birmingham, Birmingham, AL USA

## Abstract

In eukaryotes, DNA is packaged into chromatin, which presents significant barriers to transcription. Non-histone chromatin proteins such as the Heterochromatin Protein 1 (HP1) proteins are critical regulators of transcription, contributing to gene regulation through a variety of molecular mechanisms. HP1 proteins are highly conserved, and many eukaryotic genomes contain multiple HP1 genes. Given the presence of multiple HP1 family members within a genome, HP1 proteins can have unique as well as shared functions. Here, we review the mechanisms by which HP1 proteins contribute to the regulation of transcription. Focusing on the *Drosophila melanogaster* HP1 proteins, we examine the role of these proteins in regulating the transcription of genes, transposable elements, and piRNA clusters. In *D. melanogaster*, as in other species, HP1 proteins can act as transcriptional repressors and activators. The available data reveal that the precise impact of HP1 proteins on gene expression is highly context dependent, on the specific HP1 protein involved, on its protein partners present, and on the specific chromatin context the interaction occurs in. As a group, HP1 proteins utilize a variety of mechanisms to contribute to transcriptional regulation, including both transcriptional (i.e. chromatin-based) and post-transcriptional (i.e. RNA-based) processes. Despite extensive studies of this important protein family, open questions regarding their functions in gene regulation remain, specifically regarding the role of hetero- versus homodimerization and post-translational modifications of HP1 proteins.

## Introduction

Variation in chromatin structure influences transcriptional regulation [1], and non-histone chromosomal proteins such as the Heterochromatin Protein 1 (HP1) family play an important role in this process. The fundamental unit of chromatin is the nucleosome, an octamer containing two copies each of histones H2A, H2B, H3, and H4 [2]. Histone variants can replace the core histones under specific circumstances, contributing to transcriptional regulation [3, 4]. Histone proteins have disordered tails that can be post-translationally modified [5]. The repertoire of histone tail modifications is diverse, including for example methylation, acetylation, phosphorylation, ubiquitylation, crotonylation, and GlcNAcylation [6–8]. Many non-histone chromatin proteins can be classified into three groups based on their relationship to these histone modifications [9]. “Writers” modify histone proteins with novel modifications, while “erasers” remove such modifications. “Readers” recognize histones that are post-translationally modified, and HP1 proteins, which are the focus of this review, are reader proteins. Variation in histone modifications and non-histone chromosomal proteins yields rich diversity in chromatin structure, which can be classified by its composition [10]. These chromatin types often are shared between species [10], but they differ in their properties such as biophysical compaction, replication timing, and repetitive DNA content and have significant impacts on transcriptional regulation.

**Fig. 1:**
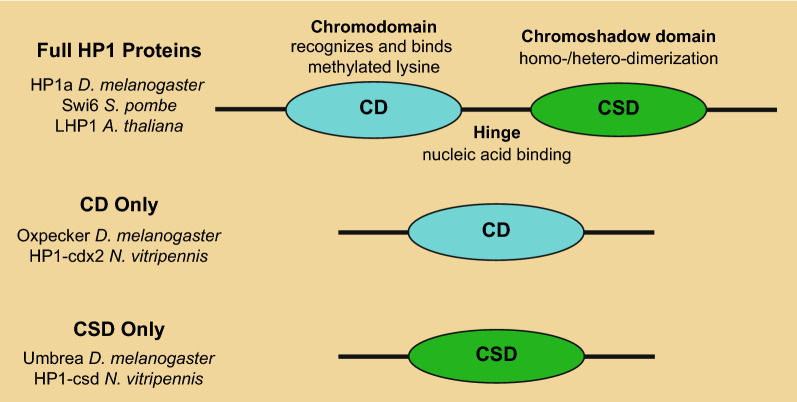
Structure of HP1 orthologs and related proteins. To be classified as a full-length HP1 family member, a gene must code for a chromodomain (CD), a chromoshadow domain (CSD), and a hinge region. The CD mediates binding to methylated lysine residues on histone tails and in other proteins. The CSD mediates formation of homo- and heterodimers. Examples of full-length HP1 proteins include HP1a in *D. melanogaster,* Swi6 in *S. pombe,* and LHP1 in *A. thaliana.* Partial duplications of HP1 protein encoding genes result in descendant genes that contain either only a CD (such as *D. melanogaster Oxpecker*) or only a CSD (such as *D. melanogaster Umbrea*). Typically, the proteins derived from these partial duplications are classified as “HP1-like” [11]

The HP1 family is a highly conserved group of non-histone chromosomal proteins implicated in diverse nuclear processes including transcriptional regulation [11, 12]. HP1 family members are defined by their structure (Fig. 1) [13]. HP1 proteins contain an amino-terminal chromodomain (CD), responsible for the recognition and binding of methylated histone tails (hence their classification as reader proteins) [14], and a carboxyl-terminal chromoshadow domain (CSD), responsible for mediating homo- and hetero-dimerization [15]. The two regions are connected by a hinge domain which confers nucleic acid binding activity [16]. Additionally, HP1 proteins may contain N-terminal intrinsically disordered tails of variable length, although this property is not a requirement for classification as an HP1 protein, and some HP1 proteins lack these tails [17]. While the functions of some HP1 proteins (e.g., *S. pombe* Swi6 [18, 19]) are well characterized, how HP1 proteins in general impact gene regulation in the context of chromatin is not well understood.

Chromatin poses many barriers to transcription, and the presence or absence of histone modifications and non-histone chromosomal proteins determines how strong or weak this barrier is. Nucleosomes limit the accessibility of regulatory DNA sequences and block the elongation of polymerases in gene bodies [20]. In order for transcription to occur, *cis*-regulatory elements and gene promoters must be accessible for RNA polymerase and its accessory factors to be recruited, and the polymerase complex must be able to elongate through the gene body [1]. Therefore, transcriptional activity requires coordinated chromatin changes precipitated by writer, eraser, and reader proteins, as well as by chromatin remodelers which can move nucleosomes [21, 22]. While the contribution of many individual non-histone chromosomal proteins to transcriptional regulation is clear (e.g., histone acetylases and histone deacetylases), there remain a significant number of chromatin proteins, for example the HP1 family, where such an understanding is lacking. Currently, it is unclear why HP1 family members, despite a shared and highly conserved protein structure, can sometimes function as transcriptional activators, sometimes as transcriptional repressors, and sometimes, both functions are reported for the same protein. Deepening our understanding of how the presence of HP1 proteins changes chromatin structure, modifies the chromatin barrier to transcription, and contributes to transcriptional regulation is essential to understanding this important protein family.

In this review, we examine the role of HP1 proteins in transcriptional regulation. Given the diverse transcriptional impacts reported for HP1 proteins, we examine the roles of individual HP1 proteins in transcriptional regulation using Drosophila as a model, supplemented with data from other species as appropriate. We find that a subset of HP1 proteins regulate transcriptional activity by the formation of repressive chromatin domains, while other impact transcriptions through roles in co-transcriptional splicing and interactions with RNA binding proteins. Some HP1 proteins are involved actively in both mechanisms, challenging the common assertion that individual HP1 proteins are either ‘repressors’ or ‘activators’ of transcription. The available data suggest that for these HP1 proteins with dual functions in gene regulation, the specific effect on transcription at a given locus strongly depends on the protein partners, including other HP1 proteins, that are present. Our review focused on HP1 proteins highlights the diverse and multi-faceted impacts of this group of non-histone chromosomal proteins on transcriptional regulation and draws attention to topics that require further study.

## Evolutionary turnover of HP1 genes creates potential for functional diversity

**Fig. 2:**
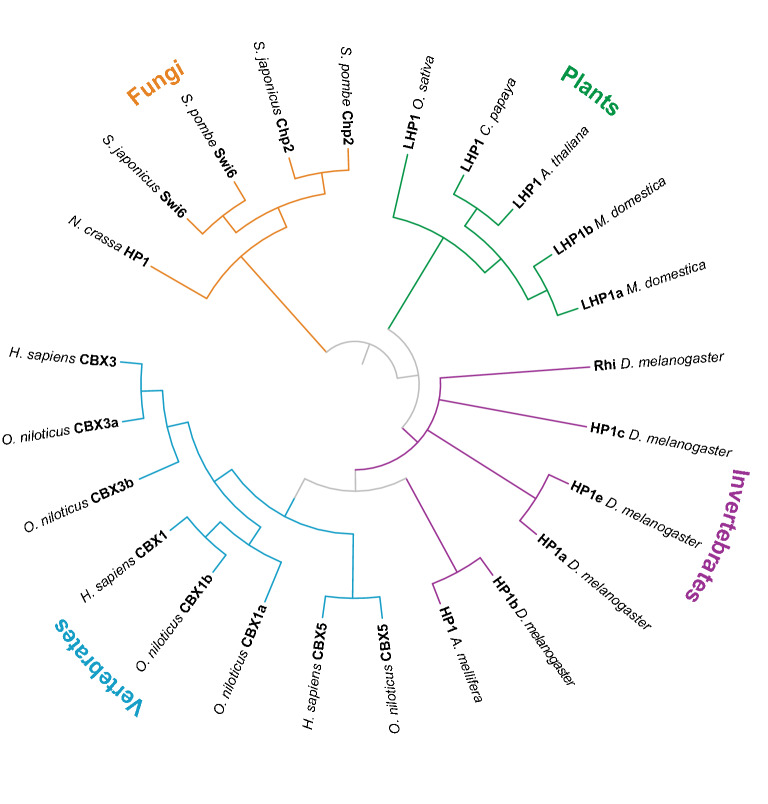
Simplified phylogeny of the HP1 family in eukaryotes. The HP1 family is highly conserved, but many genomes contain multiple orthologs. In fungi (orange), two HP1 orthologs have evolved over time with divergence in function: Swi6 and Chp2 (shown here for *Schizosaccharomyces pombe* and *S. japonicus* as well as the outgroup *Neurospora crassa*). In plant genomes (green) LHP1 orthologs evolve monophyletically (shown here are genes from *Arabidopsis thaliana*, *Oryza sativa*, *Carica papaya*, and *Malus domestica*). In invertebrates, the *D. melanogaster* genome has five orthologs which are conserved to varying degrees across Diptera. One of these genes, *HP1b*, is most closely related to both HP1 orthologs in other invertebrates (such as hymenopteran insects like *Apis mellifera* shown here) as well as vertebrate HP1 orthologs from *Homo sapiens* and *Oreochromis niloticus* (blue). This phylogenetic tree has been compiled based on information in available in the literature; branch lengths are arbitrary

HP1 proteins tend to present in genomes as small gene families, the size of which can vary extensively (Fig. 2). The presence of multiple HP1 paralogs in a single genome and subsequent specialization in function is well-illustrated in the genome of the budding yeast *Schizosaccharomyces pombe.* Here, Swi6 was first identified as a regulator of meiotic recombination and transcription of the mating type locus [23–27] and later recognized as an HP1 family member through sequence similarity [27]. Besides *Swi6*, the *S. pombe* genome contains one other HP1 family member*, Chd2* [28]. Chd2 also is involved in transcriptional silencing but is nonetheless functionally distinct from Swi6 as these proteins are unable to compensate for each other [29, 30]. They participate in different protein complexes, and their interactions with chromatin exhibit distinct kinetics [29, 30]. Thus, the *S. pombe* genome demonstrates the evolution and specialization of HP1 genes in a simple case when only two paralogs are present, a principle that can be extended to other genomes with larger HP1 gene families.

Historically, *Drosophila melanogaster* has been an important model for the study of HP1 proteins, as the gene family was first discovered in this species. The first HP1 protein to be described in *D. melanogaster* was HP1a, which is well-known for its essential roles in heterochromatin function. It was identified by producing monoclonal antibodies against proteins isolated from fractionated nuclei originating from Drosophila embryos [31] and subsequently characterized by its molecular weight, localization to heterochromatin, and cDNA sequence. Following the completion of the first *D. melanogaster* reference genome assembly, HP1b and HP1c were discovered through sequence similarity searches in 2001 [32], and two germline-specific HP1 proteins, Rhino (also known as HP1d) and HP1e were described shortly thereafter [33, 34]. Later comparative phylogenomic analyses showed that Dipteran HP1 genes evolve rapidly and arise de novo from duplication events [11, 17]. For example, over 250 million years of evolution in the Diptera, 61 gene ‘birth’ events (most likely via duplication) and 9 ‘loss’ events of full-length HP1 genes (CD and CSD) were recorded, as well as an additional 60 gains of novel genes and 10 losses of existing HP1-like genes (either CD or CSD only, Fig. 1) [17]. This rate of gene gain and loss is unusual compared to other CD containing proteins [17], and phylogenomic analyses focused on hymenopteran insects [35] and fish [36] find a lower rate of gene gain and loss in these lineages. While the evolutionary turnover in the HP1 genes in the Diptera might be unusual, the five member HP1 gene family in *D. melanogaster* can serve as model to understand the functions of HP1 proteins in gene regulation.

Given their propensity to form small gene families, the relationships between HP1 genes in different species are not always obvious. Phylogenomic analyses have shown that *D. melanogaster* HP1b most closely resembles the ancestral metazoan HP1 protein, as it is most closely related to hymenopteran HP1 proteins [35] and vertebrate HP1 proteins [11]. Mammalian genomes have expanded to contain three HP1 orthologs, all most closely related to *D. melanogaster* HP1b: HP1α, HP1β, and HP1γ, with the official names CBX5, CBX1, and CBX3 in human (Fig. 2, blue) [11]. Of these, HP1α and HP1β are enriched in the heterochromatic compartment of the genome, while HP1γ is enriched throughout both heterochromatin and euchromatin [37]. Fungal genomes tend to contain fewer HP1 genes (Fig. 2, orange). As discussed earlier for *S. pombe*, in fission yeasts, there are two HP1 family lineages, *Swi6* and *Chp2* [23, 28], that both have distinct functions in the formation and structure of heterochromatin [29]. The genome of the bread mold *Neurospora crassa* contains a single HP1 protein, and the genome of the budding yeast *Saccharomyces cerevisiae* contains no HP1 genes [11] (here, protein members of the Silent Information Regulator complex form heritable repressive chromatin [38]). Plant-specific Like-Heterochromatin Protein 1 (LHP1) genes (Fig. 2, green) and Hhp1p from *Tetrahymena thermophila* represent distinct HP1 gene lineages with functions different from animal and fungal HP1 proteins [39, 40]. While HP1 paralogs in fungal and animal genomes typically recognize di- and trimethylation of H3 lysine 9 (H3K9), these gene products recognize H3 lysine 27 (H3K27) methylation, which is more commonly recognized by another CD-containing protein, Polycomb. Phylogenetic analyses suggest that LHP1 genes share a single shared common ancestor with animal and fungal HP1 genes, later followed by duplication and expansion of LHP1 genes in monophyletic branches resulting in the presence of multiple LHP1 genes within individual genomes [41, 42]. Together, the comparative sequence analyses from animal, plant, and fungal genomes confirm that HP1 genes are encoded by small gene families across eukaryotes.

Overall, surveying HP1 paralogs across eukaryotic genomes reveals a consistent pattern of diversification. HP1 genes are duplicated, and ancestral functions are subdivided (sub-functionalization) or they assume novel biological functions (neo-functionalization) if retained in the genome. Common themes observed include the expression of evolutionarily young genes within the germline in animals, and a tendency to be enriched in repressive chromatin domains. However, in several lineages, individual HP1 family members occasionally have evolved independently to localize to euchromatin instead. The *D. melanogaster* genome encodes both younger, germline expressed HP1 proteins as well as older proteins with enrichment in both euchromatin and heterochromatin. Thus, *D. melanogaster* can help us understand the extent to which these proteins exhibit functional diversity, and potentially collaborate, in the regulation of gene expression.

## HP1a is a critical heterochromatin component with both repressive and activating functions in transcriptional regulation

**Fig. 3:**
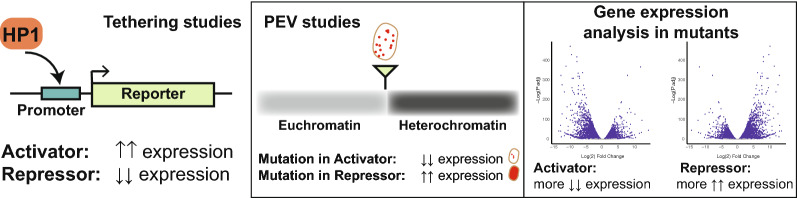
Assays typically used to determine if HP1 proteins are repressors or activators. There are three assays used most often to determine if a protein functions as a transcriptional activator or repressor: (1) Tethering studies, (2) position effect variegation (PEV) studies, and (3) expression studies in mutants. Tethering studies (left) use various methods (e.g. CRISPR/dCas9, LacI/LacO, etc.) to recruit a protein of interest to the promoter of a target reporter gene to observe if gene expression is increased, suggesting the protein is an activator of transcription, or decreased, suggesting the protein is a repressor. PEV studies (middle) utilize reporter genes with varying expression, shown here for the *white* gene in fly eyes and introduce a mutant allele for the protein of interest. If the protein of interest is a transcriptional repressor or functions in the maintenance of heterochromatin, a mutation will increase expression of the variegating reporter. If the protein of interest is a transcriptional activator or counteracts heterochromatin formation, a mutation will decrease expression of the variegating reporter. Gene expression studies in mutants (right) suggest that the protein functions as an activator is most genes are downregulated compared to wildtype, while if the protein functions as a repressor, most genes will be upregulated compared to wildtype

**Fig. 4:**
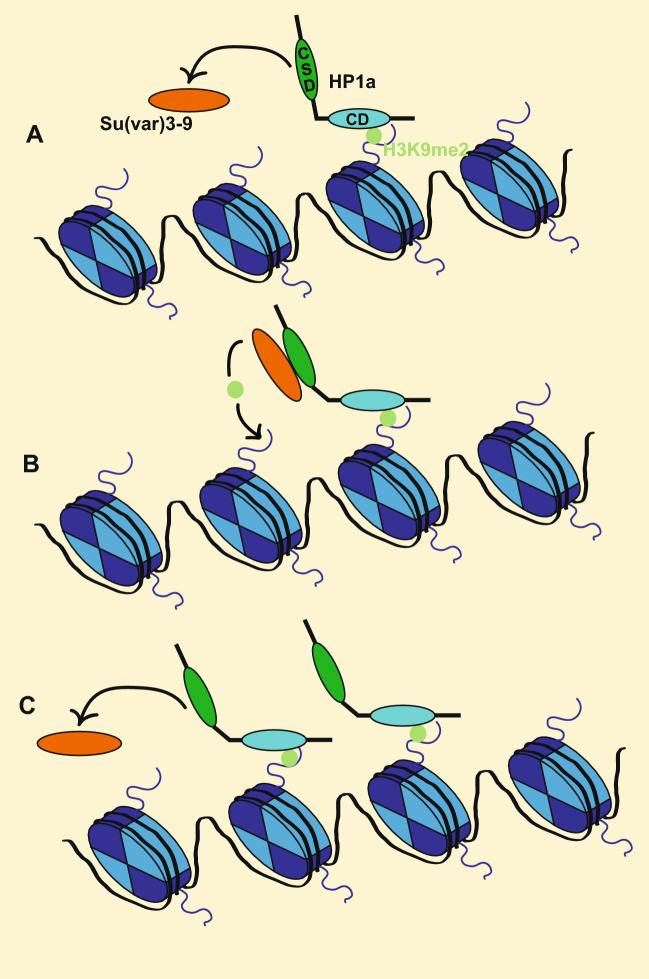
Self-propagation of heterochromatin by the interaction of HP1a and Su(var)3-9. **A** The Drosophila HP1 ortholog HP1a recognizes and binds H3K9me2/3 (light green) through its CD (green oval). It subsequently recruits the H3K9 methyltransferase Su(var)3-9 (orange oval) to these loci through its CSD (teal oval). **B** Once recruited by HP1a, Su(var)3-9 deposits H3K9me2/3 modifications on nearby nucleosomes (blue). **C** The newly deposited H3K9me2 modifications serve as novel binding sites for additional HP1a proteins. Binding of HP1a at these tails subsequently results in further recruitment of Su(var)3-9, and the process continues. Propagation of HP1a results in formation of heterochromatin and compaction of chromatin structure

*Drosophila melanogaster* HP1a, the first HP1 protein to be discovered, was described initially as functioning in transcriptional repression. After its initial discovery, HP1a was characterized functionally in screens to identify modifiers of position effect variegation (PEV) (reviewed in [43]). PEV studies with multiple reporters (Fig. 3) identified the gene encoding HP1a, *Su(var)205*, as a component of heterochromatin and transcriptional repressor: Loss of HP1a resulted in de-repression of silencing and increased transcription from the PEV reporter visible by a shift from variegating to red eyes [43–47]. Later studies demonstrated that HP1a binds the repressive histone modification H3K9 di- and trimethylation through its CD and recruits Su(var)3–9, a histone methyltransferase that produces this modification, through its CSD [48, 49]. Su(var)3–9 subsequently methylates neighboring nucleosomes, increasing the number of HP1a binding sites locally, and thus the number of Su(var)3-9 binding sites [48, 49]. In this manner, these proteins propagate heterochromatin along the chromatin fiber (Fig. 4). Thus, the data from PEV studies as well as its binding to H3K9me2/3, its interaction with Su(var)3-9, and its localization to heterochromatic portions of the genome support HP1a’s function as a transcriptional repressor.

Biophysical studies have added another dimension of understanding to the function of HP1a in transcriptional repression. HP1a—as do other HP1 proteins—dimerizes via its CSD. Data from the *S. pombe* HP1 homolog Swi6 demonstrate that interactions mediated by the CD can lead to the formation of higher order polymers [50, 51], thus compacting the chromatin and likely increasing the barrier to transcription. Strom et al. proposed HP1a as a driver of liquid–liquid phase separation to split heterochromatin and euchromatin into different nuclear compartments [52]. Purified HP1a has the propensity to form liquid-like droplets in vitro, and it forms liquid-like droplets in vivo in Drosophila embryos coincident with the timing of the establishment of heterochromatin. Furthermore, Strom et al. observed decreased rates of protein diffusion across the heterochromatin–euchromatin border, consistent with differences in biophysical phases across these compartments [52]. HP1 paralogs in other species also have a propensity to form liquid-like condensates, most notably HP1α (human) and Swi6 (*S. pombe*) [53, 54], indicating this property is conserved. NMR (nuclear magnetic resonance) spectroscopy shows that upon binding of yeast Swi6 to methylated histone tails, the nucleosome core shifts, both becoming more accessible and increasing contacts with nearby nucleosomes [54]. Studies of nuclei from mouse rod cells with “inverted” nuclear architecture (heterochromatic domains in the interior instead of at the nuclear periphery) combining Hi-C mapping of 3D genome interactions with microscopy and polymer simulations found that only simulations with strong attractive forces among heterochromatin domains could replicate the observed organization of both conventional and inverted nuclei [55]. Likely, the biophysical properties of HP1a and its relatives Swi6 (*S. pombe*) and HP1α (human/mouse), their ability to compact chromatin and propensity for phase separation, contribute to their repressive effects on transcription.

Tethering studies that bring HP1a to reporter genes or other target loci further support a role for HP1a as repressor as they often report decreased expression upon HP1a recruitment (Fig. 3). Seum et al. used a *lacZ/white* transgene with GAL4 binding sites to recruit a GAL4-HP1a fusion and detected repression of *white* expression and enhanced variegation of the reporter [56]. Studies using the *lacI/lacO* system, recruiting an HP1a-lacI fusion to a reporter gene with l*acO* repeats in its promoter, found that at 25 of 26 genomic locations tested, HP1a recruitment was able to silence the *white* reporter gene, demonstrating a repressive effect of HP1a binding compared to GFP binding [57]. Similar results are reported by Lee et al. [58], and when dCas9 systems are used to bring HP1a to diverse promoter regions in the *D. melanogaster* genome: gene expression tends to be lower than without the presence of HP1a (Schoelz et al., unpublished). However, these results tend to be more variable than what is seen in the reporter genes: the gene expression impact is not nearly as uniform, and more variable responses are seen. Repressive effects are seen also when mammalian HP1α or HP1β or *Neurospora crassa* HP1 are recruited to sites of interest [59–62]. Together, tethering studies mainly support a role for HP1a as a repressor in the limited genomic contexts studied.

Gene expression changes induced by HP1a loss in *D. melanogaster* provide further insights into HP1a’s role in transcriptional regulation (Fig. 3). Gene expression changes upon knockdown include both direct effects and indirect effects (e.g., direct impacts on a transcriptional regulator and indirect impacts caused by the change in the transcriptional regulator). Thus, by themselves, such gene expression changes are not sufficient evidence for a protein’s role in gene regulation. However, these data provide important initial clues for follow-up studies. In HP1a mutants, transposable elements (TEs) within heterochromatin were shown to be upregulated [12]. Gene expression array and RNA-seq studies of HP1a mutants or HP1a knockdown in cells supported this finding and provided additional insights into HP1a’s role in gene regulation. These genome-wide studies identified hundreds of transcripts mis-regulated with loss of HP1a. While TEs were clearly upregulated, the impact on genes was more complex. For example, Cryderman et al. identified 284 upregulated and 261 downregulated genes in *Su(var)205* (the gene encoding HP1a) mutant larvae [63], while de Lucia et al. found ~ 400 genes downregulated and ~ 120 upregulated after removal of HP1a by RNAi in Kc cells [64], and Lee et al. identified 326 upregulated and 956 downregulated genes after removal of HP1a by RNAi in S2 cells [65] (all studies using microarrays). Studies using RNA-seq also find both up- and downregulation: In mutant larvae lacking HP1a, 60% of misregulated genes were downregulated [66], and in stage 14 eggs from animals depleted for HP1a by RNAi 623 upregulated and 736 downregulated genes were found [67]. Together, these studies show that a significant portion of genes are downregulated, often more than half, suggesting an activating role for HP1a, despite its accepted role as repressor. As noted above, these results are difficult to interpret as the misregulated genes include both direct effects of HP1a loss and indirect effects of, for example, cellular stress due to the genomic instability precipitated by HP1a loss. However, several studies have expanded on these findings and confirm a role for HP1a in gene activation.

As suggested by the expression analysis of HP1a mutants, there are situations when HP1a functions as transcriptional activator. HP1a is required for the expression of heterochromatic genes as well as a subset of euchromatic genes [63, 68–70]. While this finding at first appears paradoxical given the role of HP1a in transcriptional silencing, two main mechanisms have been proposed to explain the ability of HP1a to positively regulate gene expression: maintenance of heterochromatin structure and facilitating transcriptional elongation [13]. First, consider the function of HP1a in positive regulation of heterochromatic genes. Drosophila genes residing within pericentric heterochromatin or on chromosome four require the presence of HP1a to be transcribed [66, 71, 72]. This requirement was first observed in early studies of PEV (Fig. 3) that measured expression of genes residing within the heterochromatin side of the heterochromatin–euchromatin border (as opposed to the euchromatin side, such as *white* described above) [71, 73]. PEV modifying mutations have the opposite effect on expression of heterochromatic genes compared to their effect on euchromatic genes [74]. Later genomic studies showed large scale repression of heterochromatic gene expression following HP1a depletion, and HP1a has been shown to promote open chromatin at these regions [66, 75]. At transcribed heterochromatic genes, HP1a is absent at the promoter, but enriched over the gene body [66, 75]. Why HP1a enrichment over the gene bodies of these genes is required for proper expression and why expression of these genes is misregulated when they are translocated to euchromatin is not known. It has been speculated that heterochromatin genes have adapted to this distinct chromatin environment, but further studies testing this hypothesis are needed.

Further, HP1a also positively regulates expression of euchromatic genes [76], and it is involved in the induction of heat-shock genes [70]. Piacentini et al. found that Drosophila HP1a interacts directly with RNA polymerase II as well as heterogenous nuclear ribonuclear proteins (hnRNPs), suggesting a role for HP1a in RNA processing. Interestingly, hnRNP interacting partners were observed to be suppressors of PEV, suggesting a role in heterochromatin structure [69]. A similar role for regulation of RNA processing has been observed for HP1γ [77] and also specifically for the *Sxl* locus in *D. melanogaster*, where HP1a loss cause splicing defects [78]. While this finding suggests a connection between HP1a’s functions in RNA processing and heterochromatin function, it is important to note that the targeting of HP1a to actively transcribed genes is independent of its targeting to H3K9 methylation [79]. Thus, HP1a’s function in transcriptional activation appears different from its function in heterochromatin formation and transcriptional repression, possibly depending on interacting partners, but many details of its role in transcriptional activation remain to be uncovered.

## Evidence for HP1b as a transcriptional silencer and activator

Drosophila HP1b has a complex evolutionary history which is relevant to understanding its functions in transcriptional regulation. HP1a has a conserved function that is shared with many HP1 orthologs in other species, namely its essential roles in the formation of heterochromatin at centromeres and telomeres. While this specific function of HP1 proteins is conserved, based on comparative sequence analysis, HP1b shares most similarity with mammalian HP1 proteins, and thus is most similar to the ancestral HP1 gene [12]. In their phylogenetic analysis of HP1 orthologs across Diptera, Helleu and Levine hypothesize that HP1a may have usurped HP1b’s original function [17]. This interpretation is supported by the fact that while HP1a is essential for viability, HP1b is not; HP1b loss is survivable [80, 81], even though HP1b is an evolutionarily older gene, which tend to be more likely to encode essential functions. Thus, investigating the gene regulatory functions of HP1b is of interest, given its higher sequence similarity with HP1 proteins in other lineages.

In contrast to HP1a, the role of HP1b in gene regulation is not as well understood. Different lines of evidence point to functions for HP1b as either a transcriptional repressor or activator. Mills et al. analyzed the function of HP1b in vivo through the study of null alleles where portions of the *HP1b* gene were deleted [80]. First, the authors examined whether HP1b loss modified PEV (Fig. 3) using six reporters in different genomic locations (five for the *white* gene and a variegating allele of *Stubble*). The *HP1b* null alleles lead to increased silencing of the reporters in males (classifying *HP1b* as an E(var) gene), suggesting that it functions as a transcriptional activator. Profiling genome-wide expression changes in *HP1b* mutants, Mills et al. find that a majority of differentially expressed genes in third instar larvae homozygous for one of these mutations were upregulated (85%), which contrasting with the PEV assay results, would suggest that HP1b primarily functions as a repressor. However, many of the misregulated genes were not direct binding targets of HP1b and thus appear to be due to indirect effects of *HP1b* depletion [80]. Finally, a silencing function for HP1b is supported by studies tethering HP1b to a *white* reporter gene, which results in subsequent silencing of the reporter visible through reduced eye pigment [82], confirmed independently by Lee et al. [58]. Thus, the available evidence suggests that, like HP1a, HP1b appears to be capable of both transcriptional repression and transcriptional activation, but it is much less clear how and under which circumstances HP1b might bring about these different transcriptional outcomes.

HP1b’s genome-wide binding patters shed some light on the seemingly contradictory evidence regarding its role in transcription. When they were originally described, Drosophila HP1a was described as a heterochromatin protein, HP1c was described as having a euchromatic localization, and HP1b was described as localizing to both compartments [32]—and thus often ignored. Detailed mapping of HP1a, HP1b, and HP1c genome-wide with ChIP-seq and similar methods has revealed that, while there are biases, all three HP1 proteins occur in both chromatin compartments, with more than 90% of HP1b enriched regions being in euchromatin (similar to HP1c discussed below) [83]. Furthermore, there is significant overlap in binding sites of these proteins. Specifically, both HP1b and HP1c bind throughout heterochromatin and euchromatin and share a majority of their binding sites in multiple cell types. For example, in S2 cells, 64% of HP1b enriched regions are shared with HP1c, and 89% of HP1c enriched regions are shared with HP1b [83]. HP1b and HP1c also share interacting protein partners [84]. Studying the interactomes of HP1a, HP1b, and HP1c by MudPIT, Ryu et al. found a high degree of overlap: Among 64 HP1b interactors and 43 HP1c interactors, 29 interacting proteins are shared between HP1b and HP1c [84]. This high degree of overlap in binding sites between HP1b and HP1c as well as the partial overlap in interacting proteins might explain why the finding regarding HP1b’s role in gene regulation are complex: it is possible that HP1b enrichment has different gene regulatory impacts depending on if its binding by itself or together with HP1c. Our recent analysis suggests that taking into account the combination of HP1 proteins present at a locus in *D. melanogaster* might indeed lead to a better understanding of the transcriptional outcome [83]. Additional studies perturbing individual HP1 proteins and investigating the impact on the others would be helpful to gain further insights into the coordinated roles of specifically HP1b and HP1c in gene regulation.

## Positive regulation of transcription by the HP1c complex

Of the Drosophila HP1 family members, HP1c has the best characterized role in active transcription [82, 85, 86]. PEV screens (Fig. 3) find that HP1c loss suppresses telomere position effect, but has no effect on PEV of the *w*^*m4*^ allele [suppressed by loss of HP1a] [87]. Tethering of HP1c to a reporter gene leads to increased expression, suggesting it acts as a transcriptional activator [58, 82]. Loss of HP1c leads to somewhat more downregulated than upregulated genes, consistent with a role in activation [82, 83]. HP1c is distributed throughout the euchromatic arms of the Drosophila genome and mostly associates with promoters [32, 83]. Contrasted to HP1b, HP1c is more distinct from HP1 orthologs in vertebrate and arthropod lineages [11], and HP1c does not appear to share the conserved silencing functions of many HP1 proteins [82, 85, 86]. However, it has some similarity in function to mammalian HP1β and HP1γ, which also bind extensively throughout euchromatin [37]. Available evidence suggests that HP1c is a transcriptional activator that mediates its effects on gene expression through interactions with several binding partners, including HP1a and HP1b [86], and a recent rescue experiment demonstrates that HP1γ can rescue phenotypes of an HP1c mutant [88]. However, as with HP1a and HP1b discussed above, there is additional evidence that suggests a model with HP1c functioning solely as transcriptional activator is too simplistic.

The current model for how HP1c functions as a transcriptional activator involves two steps: 1) targeting of HP1c to active loci through interactions with zinc finger transcription factors, and 2) activation of transcription by modulation of RNA polymerase II activity [86]. Font-Burgada et al. identified a key interaction between HP1c and the zinc finger transcription factors without children (Woc) and Relative of woc (Row) [82] (describe also in 2009 by Abel et al. [89]). Co-immunoprecipitation of HP1c with either of these proteins was dependent on the PxVxL amino acid motif of the HP1c CSD. HP1c localization throughout euchromatin overlapped with the genome-wide distribution of actively transcribing RNA polymerase II. Depletion of Woc or Row via RNAi abolished recruitment of HP1c to the euchromatin. In addition, HP1b recruitment to the same areas was diminished following Woc or Row RNAi treatment [82]. Later, it was shown that HP1a, HP1b, and HP1c all interact with both Woc and Row [84]. These findings demonstrate that interactions with sequence-specific transcription factors are an alternative means for targeting HP1 proteins to chromatin independent of its binding to methylated histones [see [79] for a discussion of H3K9me-independent discussion of HP1a).

Following its targeting to gene promoters, HP1c acts to stimulate transcription by enhancing RNA polymerase II elongation through two mechanisms. First, HP1c facilitates the release of pause RNA polymerase II from the promoter, thus increasing transcription. Following the initiation of transcription at gene promoters, RNA polymerase II briefly transcribes a short RNA transcript and subsequently pauses before continuing to elongate the remaining RNA transcript [90]. Release from pausing requires phosphorylation of the carboxyl terminal disordered tail of RNA polymerase II by the kinase CDK9 [1]. The phosphorylation activity of CDK9 is connected to the deposition of monoubiquitylation on H2B by the E3 ligase Bre1 (for a recent review on RNA pol II and histone modifications, see [91]). H2B monoubiquitylation is correlated with active transcription, and in *S. pombe*, the coordinated activity of Bre1 and CDK9 represses antisense transcripts [92]. The HP1c complex recruits the ubiquitin receptor Ubqn to target gene promoters to block activity of the de-ubiquitinase Non-stop (Not), a component of the SAGA complex [93]. Specifically, Woc recruits Ubqn to gene promoters and complexes with Row, which in turn recruits HP1c. Depletion of Ubqn diminishes occupancy of RNA polymerase II as well as the elongation factor NELF at transcription start sites and results in decreased expression of HP1c target genes. Simultaneous depletion of Ubqn and Not rescues H2B-ubiquitylation, RNA polymerase II occupancy, and gene expression. Recruitment of HP1c is unaffected by the depletion of Ubqn or Not [93], but Woc and Row are needed for Ubqn and HP1c to bind to chromatin [94]. Thus, HP1c facilitates transcription by protecting H2B monoubiquitylation, which promotes transcriptional elongation.

HP1c also promotes transcription through the recruitment of the Facilitates Chromatin Transaction (FACT) complex. HP1c, and to a lesser extent HP1a and HP1b, interacts with the Ssrp1 subunit of the FACT complex [85]. FACT is targeted to actively transcribed genes and binds disrupted nucleosomes [95]. The crystal structure of FACT suggests that it mimics DNA binding to displaced H2A-H2B dimers [96]. Thus, FACT is hypothesized to preserve chromatin structure by stabilizing nucleosome intermediates at actively transcribed genes and in doing so facilitate transcription. Depletion of HP1c abolishes FACT recruitment to chromatin [85]. This disruption included reduced recruitment of FACT to heat shock loci and subsequent reduced expression of heat shock proteins during the heat shock response [85]. Thus, HP1c facilitates transcription through both preservation of monoubiquitylated H2B and the recruitment of the FACT complex. Interestingly, these two processes are dependent on each other. Monoubiquitylated H2B helps stimulate deposition of H2A-H2B dimers by FACT back into nucleosomes in actively transcribed gene bodies [97, 98]. One caveat is that HP1c targeting does not always result in activated expression. For example, targeting of HP1c to Notch target genes appears to result in the repression of these genes [88]. However, a comprehensive view of available evidence shows that HP1c promotes transcription at most of its targets by influencing multiple, synergistic processes through its interacting partners.

**Fig. 5:**
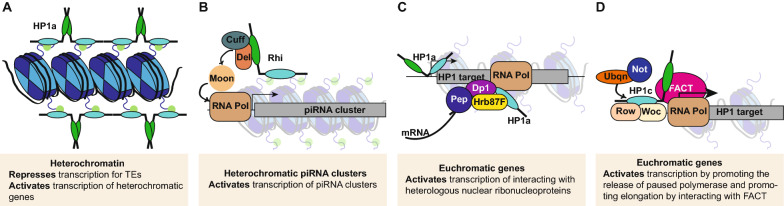
Mechanistic basis for the role of HP1 proteins in gene regulation. **A** Within heterochromatin, HP1a promotes the formation of a condensed chromatin structure by binding to H3K9me2/me3 and forming polymers that bridge between adjacent polymers. This specialized chromatin structure represses expression of TEs and reporter genes but is required for the expression of genes native to this environment. **B** At heterochromatic piRNA clusters, the germline specific HP1 homolog Rhi binds to H3K9me2/me3 and together with Del and Cuff forms the RDC complex. The RDC complex then recruits Moon, which bypasses some of the steps required in euchromatin for RNA polymerase recruitment. Moon thus allows RNA polymerase recruitment to this chromatin environment and leads to transcription of the piRNA clusters. HP1a is also present and prevents transcription from other sites not targeted by RDC and Moon. **C** At some euchromatic genes, HP1a can be found at the promoter and interacting with heterologous ribonuclear proteins. It also interacts with RNA polymerase and the mRNA that is being produced. These interactions have an activating effect on transcription at these target sites. **D** At many euchromatic genes, HP1c is found in the promoter region together with the transcription factors Woc and Row. Together, they recruit Ubqn and Not, which promote the release of paused RNA polymerase. In addition, HP1c interacts with FACT, which promotes elongation by RNA polymerase. These two processes lead to transcriptional activation

Its interaction with the insulator protein BEAF-32 might also contribute to HP1c’s role in transcriptional activation [99]. BEAF-32 binds near the transcription start sites of housekeeping genes to activate gene expression [100, 101] and HP1b and HP1c binding sites are enriched for the BEAF-32 binding motif [83]. BEAF-32 facilitates long-range physical interactions between transcription factors and promoters [102], thus promoting transcription. BEAF-32 works synergistically with the transcription factors Serendipity-δ and Row to drive expression of housekeeping genes [99, 102]. Additionally, BEAF-32 promotes long-range interactions, allowing Row to activate a set of developmental genes that lack direct Row or BEAF-32 binding sites [99]. BEAF-32 physically interacts with both HP1b and HP1c, but how these HP1 proteins influence BEAF-32 activity is unknown. Given that depletion of HP1c results in downregulation of a common set of genes compared with depletion of Woc or Row [82], it is likely that HP1c affects BEAF-32 activity in transcriptional activity. Future studies of this interaction are needed to determine whether an interaction between BEAF-32 and HP1 proteins may regulate gene expression through facilitating long-range interactions and 3D genome structure. Together, the available data support a role for *D. melanogaster* HP1c as a transcriptional activator that functions through several distinct molecular mechanisms (Fig. 5).

## Active transcription of piRNA clusters facilitated by Rhino (HP1d) and HP1a

In addition to their functions in the regulation of genes and TEs, HP1 proteins also play important roles in the regulation of piRNAs. piRNAs are small noncoding RNAs (< 30 nucleotides) that repress TEs (reviewed in [103]). Briefly, piRNAs target TEs through base pairing of complementary sequences. In Drosophila, the piRNA pathway depends on three Argonaute proteins: Piwi, Aub, and AGO3. Piwi mediates export of long piRNAs precursor transcripts from the nucleus to cytoplasmic exonucleases, which cleave the transcripts. The resulting piRNAs are bound by Aub and Ago-3, forming piRNA complexes that then recognize and cleave complementary RNA molecules, producing additional piRNAs. These secondary piRNAs create a feedback loop for recognition and repression of additional TE sequences in the genome (“ping-pong” amplification of piRNAs [104, 105]). piRNAs are inherited through the female germline, and maternal piRNAs are responsible for the initial recognition of TE sequences in the zygotic genome [103]. When maternally inherited piRNAs fail to target paternally inherited TEs not present in the maternal genome, they cannot be regulated, leading to sterility known as hybrid dysgenesis [106]. piRNAs are transcribed from so-called piRNA clusters, which contain the TEs they repress [103]. This setup means that TEs must be selectively transcribed from and repressed at the same locus, and HP1 proteins with their repressive and activating functions are essential for this process.

HP1d, also known as Rhino (Rhi), is a critical factor for the transcription of piRNA precursors from piRNA clusters [107]. Unlike Drosophila HP1a, HP1b, and HP1c which are expressed ubiquitously in somatic cells, Rhi is expressed in the female germline [33]. Rhi has a history of positive selection [34], and it localizes to piRNA clusters, targeted by a mechanism independent of piRNA production [107, 108]. Depletion of Rhi blocks the transcription of these clusters and leads to mislocalization of AGO3 and Aub [107]. H3K9 methylation produced by Eggless (Egg) facilitates targeting of Rhi to piRNA clusters through its CD [109–111]. Rhi, together with the microtubule-associating protein Deadlock and the transcription factor Cutoff (Cuff), forms the RDC complex [112–114]. Once targeted to piRNA clusters, the RDC complex potentiates transcription initiation by recruiting Moonshiner (Moon) [115]. Moon is a paralog of the basal transcription factor TFIIA, and its presence helps bypass normal requirements for initiation of transcription. Typically, initiation depends on stepwise recruitment of basal transcription factors, beginning with TFIIA and TFIID which recognize the TATA box motif within promoters [116]. However, at heterochromatic piRNA clusters the TATA box is inaccessible, and these factors cannot bind. At piRNA clusters, Moon substitutes for the TFIIA-TFIID complex, allowing for the subsequent recruitment of other basal transcription factors and eventually RNA polymerase II [115]. In addition to promoting initiation, the RDC complex also regulates splicing of piRNA precursors within the nucleus. This activity depends on the recruitment of the DEAD box protein UAP56 (known as Hel25E) to piRNA clusters [108]. Depletion of Rhi, Cuff, or Hel25E results in aberrant splicing of piRNA precursor transcripts, and tethering of Rhi to a reporter transgene suppresses splicing [108]. Interestingly, at a transgene, tethering of Rhi leads to silencing, likely post-transcriptionally by preventing pre-mRNA splicing [108]. The suppressed splicing at piRNA clusters is hypothesized to differentiate between primary piRNA precursor transcripts and mRNAs, where splicing occurs co-transcriptionally. In summary, the Drosophila HP1 protein Rhi regulates transcription of piRNA clusters by facilitating recruitment of RNA polymerase and by regulating splicing of RNA transcripts. It is another example of how HP1 proteins can function in transcriptional activation.

Besides Rhi, HP1a is also a critical factor for the regulation of transcription at piRNA clusters in Drosophila. In contrast to Rhi’s activating role, HP1a is essential for repression at these loci. At piRNA loci, HP1a functions similarly as in heterochromatin discussed earlier. HP1a interacts with Piwi via a PxVxL motif, leading to the colocalization of these proteins across the Drosophila genome [117]. Disrupting this interaction impairs the silencing activity of Piwi [117, 118], and loss of Piwi results in a loss of H3K9me3 at TE sequences [110]. Piwi recruits Panoramix (Panx), which recruits the H3K9 methyltransferase Egg [111, 119], leading to the formation of heterochromatin. However, the function of HP1a in the regulation of piRNA clusters is more involved than repression of TEs. Loss of HP1a also results in dysregulation of piRNAs and accumulation of splicing events in piRNA transcripts, demonstrating that HP1a is essential for piRNA biogenesis [120]. Possibly, similar to what is seen for genes residing in pericentric heterochromatin or on chromosome 4, HP1a establishes the repressive chromatin environment at piRNA clusters, to which they have adapted to be transcribed properly. Thus, at piRNA clusters, the activating and repressive functions of Rhi and HP1a together create an environment where a low level of piRNA pre-cursor transcription is possible, but the transcription of TEs is inhibited (Fig. 5).

## The role of HP1e is transcriptional regulation in the male germline is unknown

HP1e, the male germline-specific HP1 homolog in *D. melanogaster* is the least studied protein in the Drosophila HP1 gene family; to date, only 10 research articles mention HP1e according to FlyBase, compared to the 555 research articles that mention HP1a [121]. Most of what we know about a potential link of HP1e to gene regulation comes from a study by Levine et al. [122]. Comparing expression levels in HP1e-depleted testes to controls, approximately 700 genes were misregulated, approximately half of them upregulated, half downregulated. Interestingly, all misregulated genes in heterochromatin were upregulated, suggesting that HP1e suppresses the expression of these genes [122]. As no genome-wide enrichment patterns for HP1e are available, it is unclear which of these gene expression changes represent direct effects, and which represent indirect effects. Cytological studies suggest that HP1e localized to heterochromatin, which suggests that at least the impacts on heterochromatic genes are direct effects [122]. Thus, the available evidence suggests that in the germline, HP1e has an effect on genes within heterochromatin that is opposite that of HP1a. While the presence of HP1a is required for the expression of genes in heterochromatin, HP1e appears to repress these genes in the germline. However, given the very limited data available for HP1e, further studies are needed to clearly define its role in gene regulation and the mechanisms that are utilized.

## HP1 proteins from other species also exhibit both activating and silencing functions

As noted above, HP1 proteins with repressive functions exist in most eukaryotic lineages, but HP1 proteins with activating functions exist outside of Drosophila as well [11, 17]. Some mammalian HP1 orthologs associate with actively transcribed genes throughout euchromatin, but they appear to serve a different function from that of Drosophila orthologs at euchromatic genes (HP1b and HP1c). Rather than enrichment at TSS regions, human HP1γ binds repeat-rich intronic regions of actively transcribed gene bodies [123–125]. This difference in binding pattern is difficult to interpret given the differences in genome organization between *D. melanogaster* and humans: The mean length for introns in the *D. melanogaster genome* is 86 bp, while it is 1747 bp for the human genome [126, 127], and gene lengths and size of intergenic regions differ significantly as well. The binding of HP1γ is hypothesized to control co-transcriptional splicing of pre-mRNA based on a study of mouse cell lines [124]. Human HP1γ binding and deposition of H3K9me3 favor the inclusion of variant exons at the *CD44* gene [125]. This function, while associated with active transcription, might be similar to the general repressive functions of HP1 proteins: HP1γ and H3K9 methylation coincide, slow down RNA polymerase II processivity and through this slow-down allow for alternative splicing. Other mechanisms are employed as well; at an inducible HIV1 long terminal repeat promoter in human cells, HP1β and HP1γ regulate transcription via a switching mechanism [128]. HP1β binding at the promoter is coincident with H3K9me3 and paused RNA polymerase II, while HP1γ binding of the promoter is associated with H3K9 acetylation and phosphorylation as well as elongation by RNA polymerase II. This finding suggests HP1β and HP1γ counteract each other at promoters and supports a model where HP1β acts a transcriptional repressor. However, HP1β might also be involved in alternative splicing (it interacts with the splicing regulator ASF/SF2) (128), as might HP1α (by targeting of siRNAs to regulate alternative splicing) [129]. Thus, all three mammalian HP1 orthologs have been linked to co-transcriptional splicing, thus showing some functional similarity to *D. melanogaster* HP1c in promoting gene expression. But further complicating the relationship between these proteins and transcription is that all three have been observed to function as transcriptional repressors of euchromatic genic binding targets as well. All three orthologs interact with H3K9 methyltransferases and DNA methyltransferases to silence gene expression in human cells [130]. This finding is consistent with the data from Drosophila where HP1a, HP1b, and HP1c all appear to interact with the H3K9 methyltransferases Egg, G9a, and Su(var)3-9 [65], which have been documented to co-precipitate in mouse [131]. In summary, both mammalian and Drosophila HP1 family members are involved in both positive and negative regulation of transcription. While their functions in the negative regulation of transcription are similar, their associations with actively transcribed euchromatic genes appear more distinct and utilize different mechanisms.

## Conclusion

The HP1 family is a highly conserved group of transcriptional regulators, which has both repressive and activating functions (Fig. 5). Within species, when multiple family members are present, they tend to have both shared and unique functions. In Drosophila, HP1a, HP1b and HP1c share many of their binding targets at protein-coding genes, but these proteins are distinct in their capacity to activate and repress transcription. Similar functional diversity exists in other species as well, and generally, the transcriptional impact of HP1 proteins tends to be highly context specific. The pathways employed by HP1 proteins to impact gene regulation are similar in the different species and center on chromatin structure as well as RNA processing. However, while research over the last 30 years has provided many insights into the biological functions of HP1 proteins, predicting how and by which mechanisms an HP1 protein will impact transcription at a specific genomic locus remains an elusive goal. The available data suggest that more research into co-binding of HP1 proteins, and the impacts of homo- versus heterodimerization is needed. In addition, it is clear that post-translational modifications, and especially phosphorylation, have important consequences for the function of HP1 proteins. These findings suggest that to be able to predict how the binding of HP1 proteins to particular genomic locations impacts transcription, data are needed as to which form of a given HP1 protein is present, as well as the specific protein partners. Thus, the example of the HP1 family highlights the importance of non-histone chromosomal proteins in transcriptional regulation, and the complexities involved in trying to understand this diverse class of proteins.

## Data Availability

Not applicable.
